# Dissecting the endothelial cell landscape in meningioma: single-cell insights into *PLVAP*+ subpopulations and their role in tumor angiogenesis

**DOI:** 10.3389/fimmu.2025.1591125

**Published:** 2025-05-27

**Authors:** Liang Zhao, Hongling Jia, Zhikai Xiahou, Li Ren, Yanbing Song, Hao Xu, Zhihan Wang, Jin Xing

**Affiliations:** ^1^ Department of Neurosurgery, Shanghai Pudong Hospital, Fudan University Pudong Medical Center, Shanghai, China; ^2^ The First Clinical Medical College, Shandong University of Traditional Chinese Medicine, Jinan, Shandong, China; ^3^ China Institute of Sport and Health Science, Beijing Sport University, Beijing, China

**Keywords:** single-cell RNA-sequencing, meningioma, tumor heterogeneity, ETS1, angiogenesis

## Abstract

**Background:**

Meningioma (MEN) is one of the most common intracranial tumors, with a significantly higher incidence rate in females than in males. Although the majority of cases are benign, tumors located in complex anatomical regions or classified as atypical or malignant have a high recurrence rate, underscoring the need to optimize therapeutic strategies to improve patient outcomes. Therefore, this study utilizes single-cell RNA-sequencing technology to investigate the interaction mechanisms between endothelial cells (ECs) and meningiomas, aiming to identify potential therapeutic targets for the treatment of MEN patients.

**Methods:**

Tissue origin analysis of different EC subpopulations was performed using Ro/e preference analysis. Gene Ontology and Gene Set Enrichment Analysis were employed to enrich and identify relevant biological processes. Slingshot and CytoTRACE were used to determine the differentiation trajectories of cell subpopulations. CellChat was utilized to predict intercellular communication between EC subpopulations and meningioma cells (MGCs). The transcription factor (TF) networks of EC subpopulations were constructed using pySCENIC, and the function of ETS1 was validated *in vitro* experiments.

**Results:**

The MEN and temporal lobe tissues’ datasets were processed through quality control and screening, and dimensionality reduction clustering identified eight cell types. We found that ECs might play a role in MEN progression and further classified them into four subpopulations. Among these, the C2 *PLVAP*+ ECs were predominantly located at the later stages of differentiation in the Slingshot analysis, suggesting a critical role in MEN’s development. Cell communication analysis revealed that MGCs might stimulate ECs to secrete angiopoietin via the MDK-NCL ligand-receptor pair, promoting angiogenesis and MEN’s progression. Using pySCENIC analysis, the key TF ETS1 was identified. *In vitro* experiments demonstrated that ETS1 promoted ECs angiogenesis, proliferation, and migration, providing valuable insights for clinical strategies targeting MEN’s treatment.

**Conclusion:**

We identified a key ECs subpopulation, C2 *PLVAP*+ ECs, which was at a critical stage of MEN progression and might influence MEN development through the MK signaling pathway via the MDK-NCL ligand-receptor pair. Additionally, we discovered the critical TF ETS1 and validated through *in vitro* experiments that it promoted MEN’s progression, offering a new perspective for clinical treatment strategies.

## Introduction

Meningioma (MEN) is common intracranial tumors originating from the meninges, accounting for 39.7% of all brain tumors and 55.4% of all non-malignant brain tumors (1). According to the WHO classification, MEN is divided into three types: benign (Grade I), atypical (Grade II), and malignant (Grade III), with benign MEN being the most common (2). In recent years, with the advancement of medical technology, the success rate of treatment for this disease has improved (3), but due to the complex pathogenesis, clinical treatment still faces significant challenges (4). MEN is commonly located at the skull base, sagittal sinus, cavernous sinus, and other regions, which are adjacent to important nerves and blood vessels (5). This makes surgical operations difficult and prone to postoperative complications such as nerve damage, bleeding, or cerebrospinal fluid leakage (6). Additionally, MEN is highly vascularized, particularly in high-blood-supply areas, where surgery may involve significant bleeding, increasing the complexity of the operation and the patient’s postoperative recovery time (7). Furthermore, complete resection of larger or more invasive MEN may be restricted by surrounding critical structures, raising the risk of tumor recurrence, especially in atypical and malignant MEN (8). Due to their special location and disease mechanisms, MEN has limited tolerance to radiotherapy and chemotherapy. It is understood that there is considerable individual variation among MEN patients, with differences in age, tumor location, size, and grade significantly affecting treatment choices and prognosis (9). In conclusion, although treatment for MEN has made progress, traditional treatment methods still have many limitations, which severely affect the long-term prognosis of patients. Therefore, there is an urgent need to introduce new technologies and innovative methods to further elucidate the pathogenesis of MEN, optimize treatment strategies, and improve treatment outcomes while reducing the incidence of complications (10).

Endothelial cells (ECs) play a central role in maintaining vascular function and tissue homeostasis, and they are particularly crucial in the tumor microenvironment (TME) (11). Research on ECs in other tumors, such as breast cancer, lung cancer, and liver cancer, reveals their multiple roles in angiogenesis, tumor metastasis, immune evasion, and treatment resistance (12–14). In the TME, ECs are not only key participants in angiogenesis but also interact directly with tumor cells to form new blood vessel networks (15, 16). Tumor cells secrete factors like VEGF that act on ECs, stimulating their migration and lumen formation, thereby driving angiogenesis (17, 18). These newly formed blood vessels support tumor growth and provide pathways for metastasis. In MEN, the interaction between ECs and tumor cells is particularly significant, and the level of angiogenesis directly influences the growth rate and invasiveness of the tumor. Therefore, ECs are a key cell type in this .15recent years, with the advancements in single-cell RNA-sequencing(scRNA-seq) technology, targeted therapies may become a potential treatment strategy for MEN (19, 20). Single-cell sequencing can precisely analyze the gene expression profiles of individual cells, revealing the heterogeneity between different cells (21–23). This is crucial for understanding complex tissues, TME, and various biological processes (24). Compared to traditional bulk RNA sequencing, scRNA-seq enables the detection of low-abundance gene expression at higher resolution, which is particularly advantageous for studying low-expressed genes, rare cell populations, and genetic mutations (25, 26). Given the high heterogeneity of MEN, this characteristic aligns well with scRNA-seq technology. This technique can uncover the molecular heterogeneity of the tumor and the features of the TME, providing deep insights into key mechanisms such as angiogenesis, immune evasion, and treatment resistance (27). Through a series of analyses, it can identify key cell types, determine the evolutionary sequence of cell subpopulations, and identify important cellular signaling pathways and receptors, further enhancing our understanding of MEN heterogeneity. This may provide potential therapeutic targets for future clinical treatments of MEN, thereby improving treatment success rates for patients.

## Materials and methods

### Data collection from MEN patients

We obtained the scRNA-seq dataset GSE256490 from the Gene Expression Omnibus (GEO) database, a publicly accessible repository for high-throughput gene expression data. Since this dataset was derived from publicly available resources and does not involve any personal or identifiable patient data, it was exempt from ethical review and approval. The data is openly available for researchers to use, ensuring transparency and reproducibility in scientific studies.

### Raw data normalization and visualization

We analyzed the 10X genomic data from each sample using the Seurat package (28–30). In addition, we used the “DoubletFinder” tool to detect and remove doublet cells, ensuring data accuracy (31–33). Cells with extreme nFeature or nCount values were excluded from the study. Additionally, mitochondrial gene expression in each cell was kept below 25% of the total gene expression, and red blood cell gene expression was limited to less than 5% of the total gene expression. After filtering for high-quality cells based on these criteria, we normalized the data using the “NormalizeData” function (34–37). The top 2000 most variable genes were identified using the “FindVariableFeatures” function (38–41). We then normalized all genes using the “ScaleData” function, followed by principal component analysis (PCA). The Harmony package was employed to remove batch effects between different samples. The top 30 principal components were selected for further analysis, and Uniform Manifold Approximation and Projection (UMAP) (42–45) was used to transform high-dimensional data into a lower-dimensional 2D or 3D space, making visualization more intuitive. Cell clusters were annotated by referencing relevant literature and using the “CellMarker” database.

### Enrichment analysis and AUCell

For Gene Ontology (GO) analysis (46–50), we performed functional analysis of biological processes using the “ClusterProfiler” R package (51–53). Gene Set Enrichment Analysis (GSEA) was conducted to evaluate the expression trends within gene sets (54–57). Additionally, we used the AUCell tool to assess the activity of gene sets in the scRNA-seq data. AUCell was a computational method for evaluating gene set enrichment in single-cell transcriptome data. The “AUCell_buildRankings” function was used to evaluate the enrichment of stemness gene sets, and the gene sets were ranked according to their enrichment levels.

### Identification of cell subpopulations

We extracted ECs and re-normalized the data to identify the top 2000 most variable genes. PCA was then used to determine the major subpopulations (58, 59). Harmony package was applied to remove batch effects between cells. Cell subpopulations were annotated based on known marker genes, and the results were visualized using UMAP.

### Differential and enrichment analysis

We used the “FindClusters” and “FindNeighbors” functions in Seurat for cell clustering, followed by “FindAllMarkers” to identify differentially expressed genes (DEGs) for each cluster (60–62). Further investigation of the heterogeneity of EC subpopulations in MEN progression was conducted, and Gene Ontology Biological Process (GOBP) and GSEA were performed for enrichment analysis of the subpopulations (63).

### Cell trajectory and stemness gene analysis

To assess the differentiation status and stemness of EC subpopulations, we performed analysis using Slingshot (36, 64) and CytoTRACE (65). Slingshot was used to infer lineage trajectories of EC subpopulations during differentiation. The “getlineage” and “getCurves” functions were used to visualize the expression levels of these lineages, elucidating the differentiation trajectories of EC subpopulations. CytoTRACE was employed to evaluate the stemness of each cell subpopulation.

### Intercellular communication analysis

We utilized the “CellChat” package to visualize the intercellular communication network (66–68). The purpose of this software was to explore the modes of intercellular interactions through signal pathways and ligand-receptor pairs. The “netVisual_diffInteraction” function was used to describe the differences in communication intensity, while the “identifyCommunicationPatterns” function was applied to identify various communication patterns.

### pySCENIC analysis

Using the pySCENIC software in Python, a gene regulatory network was constructed, and the enrichment of transcription factors (TFs) and the activity of regulators were assessed (69, 70). The goal of this analysis was to identify TFs enriched in specific cell states and explore how these TFs and their regulators influence gene expression changes. Through this evaluation, we delved deeper into the mechanisms of cell state transitions and gene expression regulation.

### Cell culture

The cell culture of Human Umbilical Vein Endothelial Cell (HUVEC) was conducted utilizing ECM medium (EC Medium) at 37°C in a humidified atmosphere containing 5% CO_2_. The initial fluid change occurred 24 hours post cell attachment, with subsequent medium changes every 2 to 3 days. Passages were digested using 0.25% trypsin at a 1:3 ratio, which is advised for use within 6–8 generations to preserve the endothelial phenotype. Aseptic conditions are rigorously upheld to prevent contamination, and cell characterization is periodically verified.

### Running quantitative real-time polymerase chain reaction and data analysis

Use qRT-PCR to measure gene expression or DNA copy number after RNA extraction (71–73). RNA was extracted, reverse transcribed into cDNA, and amplified using primer and SYBR Green or probe (denaturation at 95°C, annealing extension at 60°C, 40 cycles). The Ct value (2-Δ Δ CT technique) was used to compute relative expression, which was normalized by an internal reference gene like *GAPDH*. It is sensitive, specific, and gene quantitative research-friendly.

### The test for cell viability

For the purpose of determining the vitality of the cells, the Cell Counting Kit-8 (CCK-8) was utilized (74–76). In 96-well plates, cells were planted at a density of 1×10^3 cells per well, and then they were cultivated for eight hours. Each well received a 100 µL detection reagent, which was then incubated for a duration of one hour. The absorbance at 450 nm was recorded on a daily basis for a period of four days, and growth curves were drawn by establishing a correlation between the OD450 values and the passage of time.

### Western Blot

The Western Blot is a technique that involves the separation of protein samples based on their molecular weight via the use of polyacrylamide electrophoresis (77). Following this, the samples are transferred to a hybrid membrane (blot), and finally, the target protein is specifically identified by the use of a primary antibody/secondary antibody combination (78, 79).

### Using flow cytometry to analyze apoptotic processes

Annexin V-FITC and PI labeling conducted in flow cytometry were able to identify the presence of apoptosis. The cells were stained after being washed with PBS and after being subjected to the treatment. Annexin V might be used to label early apoptotic cells, whereas PI could be used to designate necrotic or late apoptotic cells. Flow cytometry was used to examine the staining of the cells in order to differentiate between healthy cells, early apoptotic cells, and late apoptotic or necrotic cells.

### EdU analysis

In order to conduct the EdU experiment, the cells were subjected to a culture medium that included 10 µM EdU for a duration of 30 minutes to 2 hours. Following this, the cells were fixed with 4% paraformaldehyde and permeabilized with 0.5% Triton X-100. Finally, the Click reaction mixture was introduced to indicate the initiation of DNA synthesis process. Following the application of the dye, the activity of cell proliferation was monitored using a fluorescent microscope. Additionally, DAPI staining was utilized to help in the investigation.

### Analyses of migration and invasion through the transwell assays

Within a transwell chamber that had a porous membrane and a pore size of 8 m, cells were injected with the desired organism (80, 81). In order to conduct the migration experiment, cells were introduced into the upper layer of the medium, and the media that included attraction factors (such FBS) was introduced into the lower layer of the medium. In order to imitate the matrix, Matri-gel was introduced into the chamber in order to carry out the invasion experiment. After twenty-four to forty-eight hours, the cells that did not migrate were removed, the cells that had been pierced were fixed and stained, and the total number of cells that had been penetrated was tallied.

### The test for angiogenesis

Over the course of the experiment involving endothelial angiogenesis, HUVECs were cultivated for a period of forty-eight hours after being injected into Matrigel that had been brought to room temperature in preparation. Angiogenesis was induced by the addition of growth factors like VEGF, which allowed for the observation of the tubular shape of those cells. In order to test the capacity of ECs to generate new blood vessels, microscopic imaging was utilized to examine the measurements of the length and number of tubes.

### Statistical analysis

Statistical evaluations were carried out using R package and Python software. To determine the differences among different groups, we applied the Wilcoxon test and calculated the Pearson correlation coefficient (82–84). The levels of significance were categorized as follows: *P < 0.05, **P < 0.01, ***P < 0.001, and ****P < 0.0001. Non-significant differences between groups were marked with “ns”. These statistical methods and significance markers were utilized to validate the reliability of our results and strengthen the credibility of our conclusions (85).

## Results

### Single-cell sequencing analysis revealed the microenvironment landscape of MEN tissue

**Figure 1:**
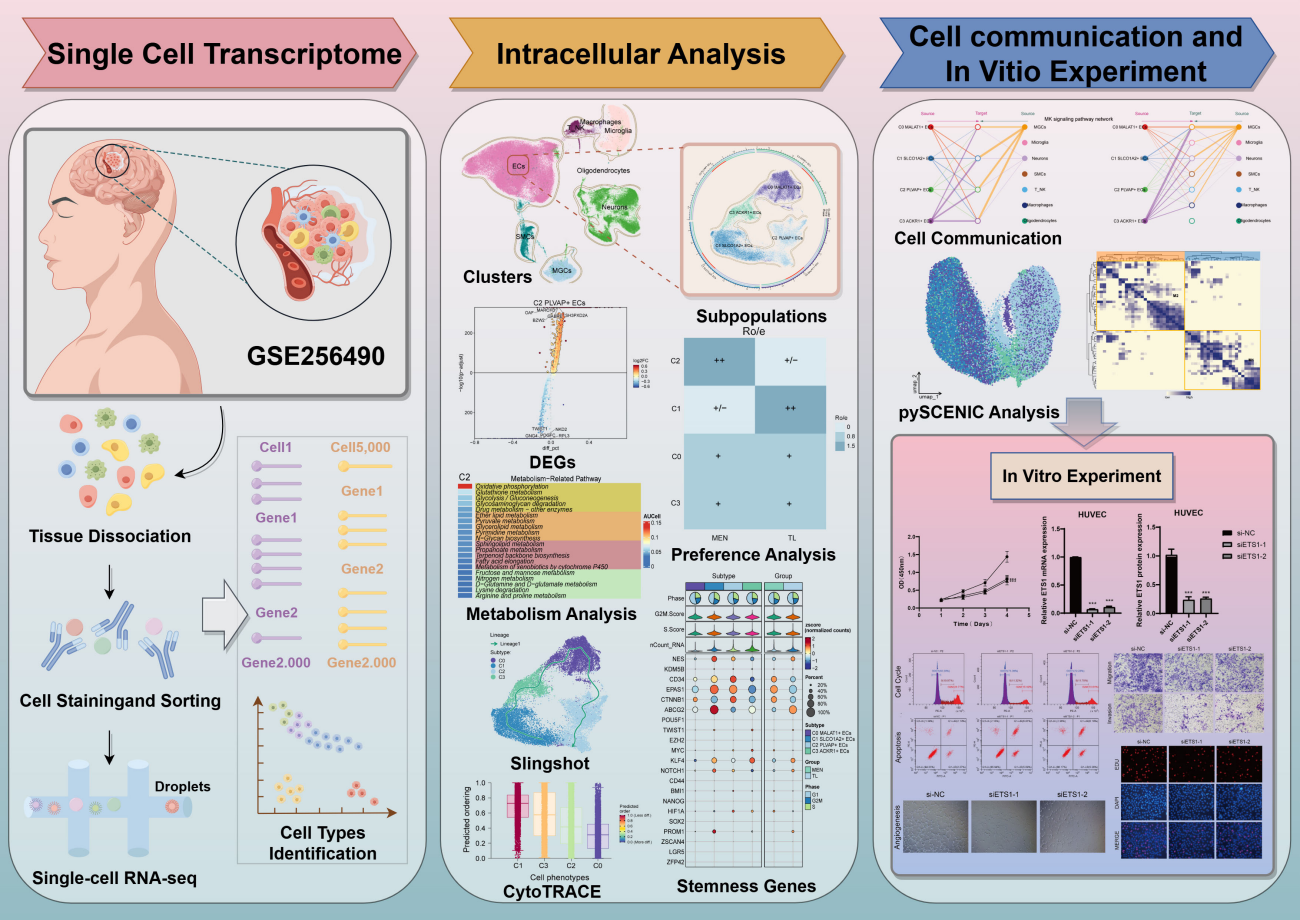
Overall workflow of scRNA-seq of MEN. Relevant sample data were obtained from the GEO database, and a series of methods were employed to analyze MEN. First of all, we used the UMAP plots to visualize different cell types and EC subpopulations. Then, methods such as Ro/e analysis, Slingshot analysis, cell stemness analysis, cell communication network analysis and pySCENIC analysis were adopted to reveal the potential pathogenesis of meningioma. Finally, *in vitro* experiments were used to verify the specific mechanism of action of the key TF ETS1.

**Figure 2:**
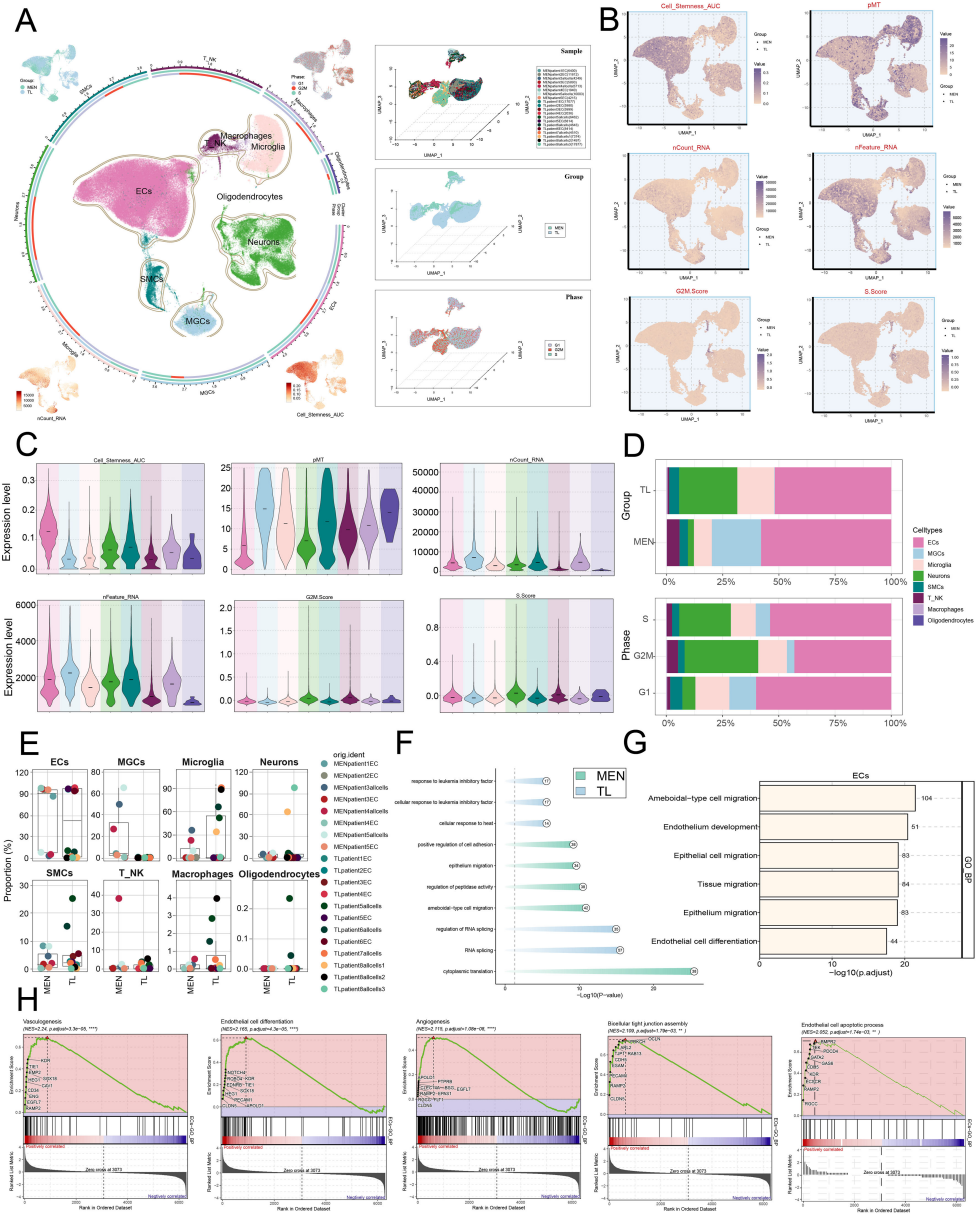
Single-cell analysis of tissue heterogeneity in MEN. **(A)** Dimensionality reduction and clustering of selected tissue samples. Eight known cell clusters were annotated. The four UMAP plots surrounding the circle, arranged clockwise from top-left to bottom-left, show the distribution features of the following: group, cell cycle phases, Cell-Stemness-AUC and nCount-RNA (left). UMAP plots displayed the distribution of sample origin, tissue classification, and cell cycle across different cell types at a three-dimensional level (right). **(B, C)** UMAP and violin plots depicted the distribution and expression levels of eight cell types across various features: Cell-Stemness-AUC, pMT, nCount-RNA, nFeature-RNA, G2/M.Score, and S.Score. **(D)** Stacked bar charts illustrated the relative proportion of groups and cell cycle phases in each cell types. **(E)** Compare the relative proportions of the eight cell types across different sample origins. **(F)** Compare the differences in enrichment analysis of different tissues. **(G)** Compare the differences in the enrichment of GOBP terms in the ECs. **(H)** GSEA enrichment analysis of GOBP terms for ECs. (**P < 0.01, ****P < 0.0001).

**Figure 1** illustrated the basic workflow of this study. We primarily utilized scRNA-seq methods, employing dimensionality reduction clustering, pseudotime analysis, metabolic analysis, stemness analysis, cell communication analysis, and TF analysis to investigate MEN. In-depth analysis was conducted to explore the specific mechanisms underlying MEN development, identify key subpopulations and target genes, and seek potential therapeutic targets for MEN treatment. To investigate the specific mechanisms underlying MEN pathogenesis and ultimately suppress disease occurrence, we conducted a single-cell analysis of MEN. First, we collected MEN and temporal lobe (TL) tissue samples. After quality control, we annotated the high-quality filtered cells into eight known cell clusters (**Figure 2A**). These clusters were identified as ECs, T cells and NK cells, macrophages, microglia, oligodendrocytes, neurons, smooth muscle cells (SMCs), and meningioma cells (MGCs). In addition, the eight clusters were further analyzed to explore sample origin, cell grouping, cell cycle, nCount-RNA, and cell stemness, providing a multidimensional understanding of the specific characteristics of cells within MEN tissues. Additionally, we used UMAP plots and violin plots to further visualize cell stemness, pMT, nCount-RNA, nFeature-RNA, G2/M.Score, and S.Score, thereby identifying the differential expression levels among different cell clusters (**Figures 2B, C**). We found that ECs exhibited the highest expression levels in stemness analysis, indicating that these cells exhibit greater differentiation potential. Next, we conducted an analysis of cell groups and the cell cycle phases (**Figure 2D**). The results showed that ECs and neurons had relatively higher proportion in the G2/M and S phases, indicating that these two cell types exhibited active DNA replication and robust proliferation. Subsequently, we conducted further visualization analysis of the sample sources for the eight known cell types to compare the proportions of different samples across different cell types (**Figure 2E**). Additionally, we analyzed the biological processes predominantly enriched in MEN cells and TL cells. MEN tissue cells were mainly enriched in “positive regulation of cell adhesion”, “epithelium migration”, “regulation of peptidase activity”, “ameboidal-type cell migration”, and “cytoplasmic translation” (**Figure 2F**). Furthermore, the GOBP terms of ECs were displayed through enrichment analysis, which revealed that ECs were primarily enriched in “ameboidal-type cell migration”, “endothelium development”, “epithelial cell migration”, “tissue migration”, “epithelium migration”, and “endothelial cell differentiation” (**Figure 2G**). By comparing the biological processes enriched in the MEN tissues and ECs, we observed certain similarities. Based on this, we hypothesized that ECs played a role in the progression of MEN. Then, to explore the role of ECs in the progression of MEN, we conducted further studies on ECs. GSEA analysis (**Figure 2H**) showed a positive enrichment trend in “vasculogenesis”, “endothelial cell differentiation”, “angiogenesis”, “bicellular tight junction assembly”, and “endothelial cell apoptotic process”. In these biological processes, ECs exhibited higher activity, suggesting that ECs might be more prominently involved in these biological functions. This also partially validated that ECs might provide essential nutritional support for the progression of MEN through vascular-related mechanisms.

### DEGs and related enrichment analysis in EC subpopulations

**Figure 3:**
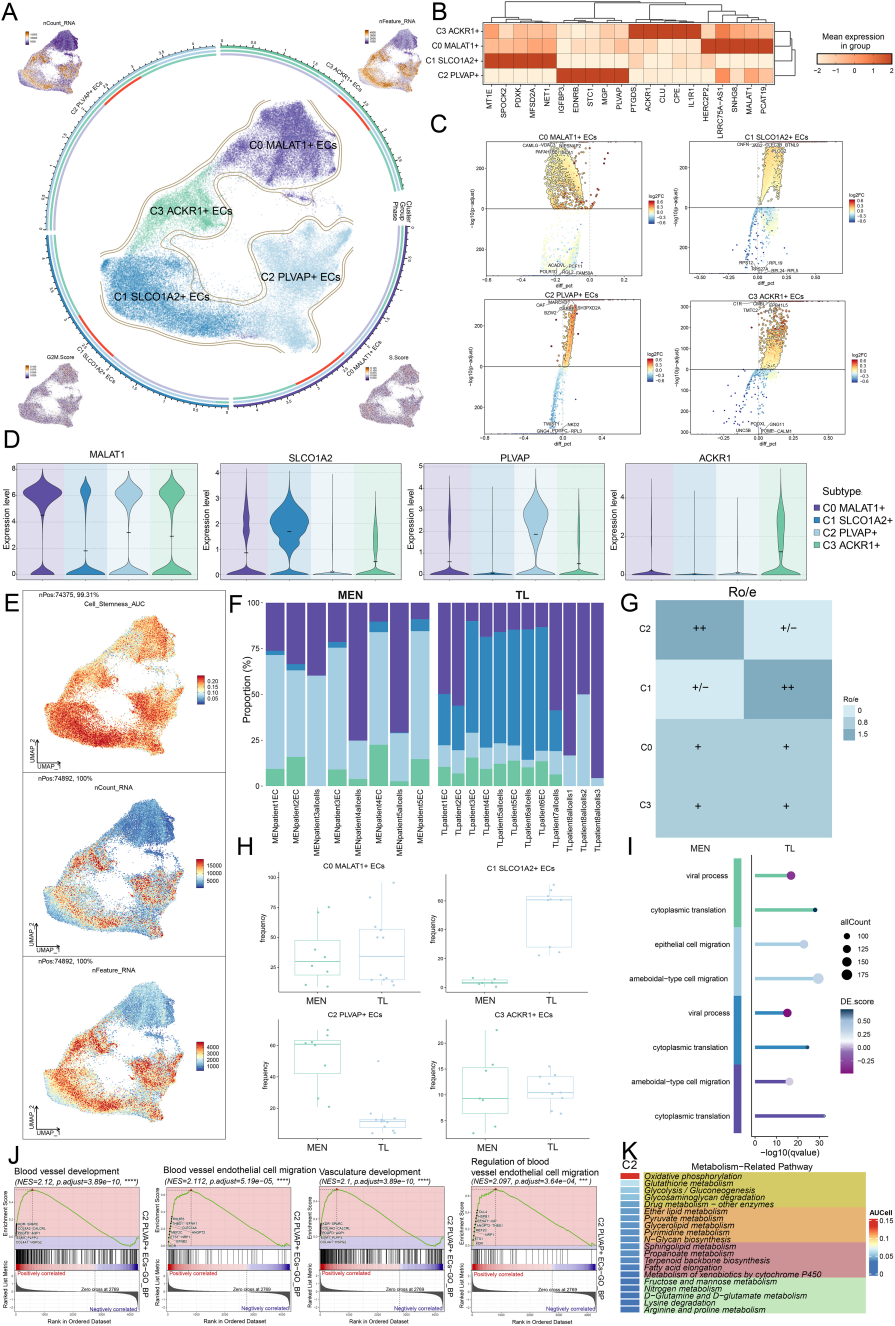
Differential enrichment analysis landscape of EC subpopulations. **(A)** Circle plot showed the distribution of EC subpopulations. The four surrounding UMAP plots displayed the expression levels of the following: nCount-RNA, nFeature-RNA, G2/M.Score, and S.Score. **(B)** Heatmap illustrated the mean expression of different genes across the four EC subpopulations. **(C)** Volcano plots showed the top 5 upregulated and downregulated DEGs in EC subpopulations. **(D)** Compare the expression levels of marker genes across EC subpopulations. **(E)** UMAP plots depicted the distribution features of EC subpopulations in relation to Cell-Stemness-AUC, nCount-RNA, and nFeature-RNA. **(F)** Stacked bar charts displayed the relative proportion of EC subpopulations in different MEN and TL tissue samples. **(G)** Ro/e analysis illustrated the preference of EC subpopulations for MEN and TL tissue types. **(H)** Box plots showed the frequency of EC subpopulations in MEN and TL tissues. **(I)** Compare the differences in the enrichment analysis of biological functions among different EC subpopulations. **(J)** GSEA enrichment analysis of GOBP terms for EC subpopulation C2. **(K)** Heatmap illustrated the top 20 metabolic pathways (AUCell values) enriched in C2 subpopulation. (***P < 0.001, ****P < 0.0001).

In the above analysis, we had identified ECs. To further clarify the role of ECs in MEN progression, we clustered the ECs into four subpopulations: C0 *MALAT1*+ ECs, C1 *SLCO1A2*+ ECs, C2 *PLVAP*+ ECs, and C3 *ACKR1*+ ECs. Additionally, the distributional characteristics of ECs subpopulations in terms of nCount-RNA, nFeature-RNA, G2/M.Score, and S.Score were visualized using UMAP plots (**Figure 3A**). We then used a heatmap to analyze the average expression levels of the top 5 genes in each subpopulations (**Figure 3B**). Furthermore, we studied the DEGs among the four subpopulations and identified 5 upregulated and downregulated genes (**Figure 3C**). To further clarify the main functions of these subpopulations, we visualized the marker genes of the EC subpopulations and displayed the results using violin plots (**Figure 3D**). In addition, we employed UMAP plots to analyze the expression levels of Cell stemness AUC, nCount-RNA, and nFeature-RNA for each subpopulation (**Figure 3E**). To explore the specific mechanisms underlying MEN development, we examined the tissue origins of each subpopulation. We found that the C2 subpopulation primarily originated from MEN tissue cells, while the C1 subpopulation predominantly came from TL tissue cells (**Figure 3F**). Compared to TL tissues, MEN tissues cells had a higher contribution to disease progression. Therefore, we hypothesized that the C2 subpopulation might play a positive role in MEN progression. Ro/e preference analysis and box plots were consistent with the above conclusions (**Figures 3G, H**). The C2 subpopulation preferred MEN tissue cells, while the C1 subpopulation preferred TL tissue cells. In subsequent analyses (**Figure 3I**), we found that the C0 subpopulation was mainly enriched in processes such as “ameboidal-type cell migration” and “cytoplasmic translation”. The C1 subpopulation was primarily enriched in processes like “viral process” and “cytoplasmic translation”. The C2 subpopulation showed significant enrichment in “epithelial cell migration” and “ameboidal-type cell migration”, while the C3 subpopulation was primarily enriched in “cytoplasmic translation” and “viral process”. Based on the comprehensive analysis of EC subpopulations, we focused on the C2 subpopulation. To further clarify the specific mechanisms of C2 subpopulation in MEN disease progression, we conducted a more detailed visualization analysis of C2 subpopulation. The biological processes enriched in the C2 subpopulation were analyzed through GSEA. The results revealed significant positive enrichment for the gene sets “blood vessel development”, “blood vessel endothelial cell migration”, “vasculature development”, and “regulation of blood vessel endothelial cell migration” (**Figure 3J**). Based on this, we hypothesized that ECs primarily promoted the development of MEN through biological processes such as angiogenesis. The relationship between ECs and angiogenesis was closely intertwined. During angiogenesis, ECs underwent proliferation and migration, providing nutrients to MEN and thereby promoting their development.

### Metabolic analysis of EC subpopulations

**Figure 4:**
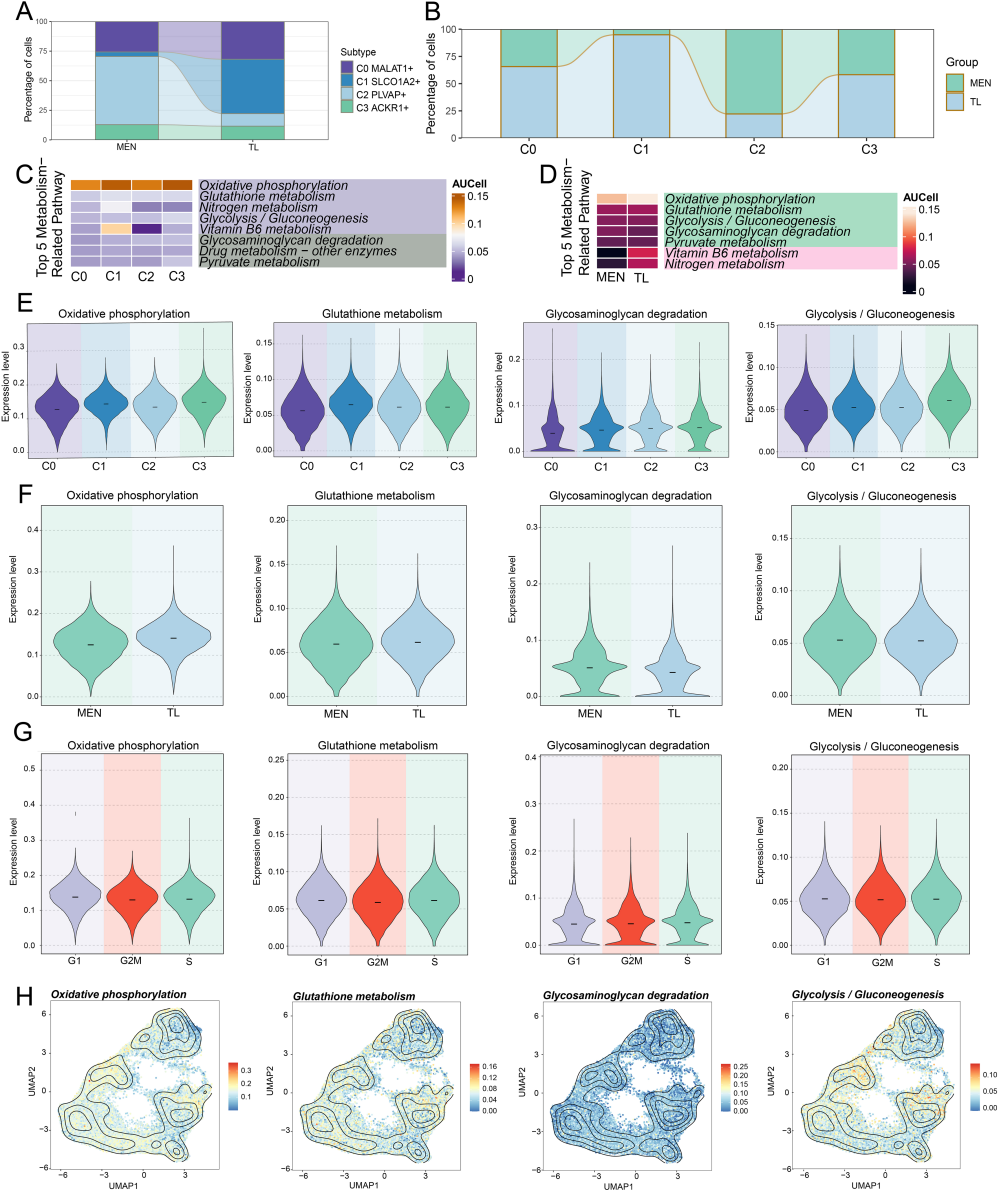
Metabolic analysis of EC subpopulations in MEN and TL tissues. **(A, B)** Stacked bar charts showed the relative proportion of EC subpopulations in different tissues (MEN and TL). **(C, D)** Heatmap displayed the top 5 metabolic pathways enriched in different EC subpopulations and distinct tissues. **(E-G)** Violin charts illustrated the expression levels of four selected metabolic pathways across different subpopulations, tissues, and cell cycle phases. **(H)** Contour plots showed the distribution features of the four selected metabolic pathways across different EC subpopulations.

To explore the specific mechanisms of EC subpopulations, we performed metabolic pathway analysis on the C2 subpopulation firstly (**Figure 3K**). It was found that the C2 subpopulation was predominantly enriched in metabolic pathways such as “oxidative phosphorylation”, “glutathione metabolism”, “glycolysis/gluconeogenesis”, “glycosaminoglycan degradation”, and “drug metabolism-other enzymes”. Besides, we conducted a comprehensive metabolic analysis of EC subpopulations. First, we analyzed the relative proportions of the four subpopulations in MEN and TL tissues from different dimensions (**Figures 4A, B**). In line with prior findings, the C2 subpopulation was primarily derived from MEN tissue cells, whereas the C1 subpopulation predominantly originated from TL tissue cells.

We then performed a comprehensive metabolic analysis of the EC subpopulations and different tissue cells. We began by enriching the top 5 metabolism-related pathways of the EC subpopulations (**Figure 4C**). Subsequently, we analyzed the top 5 metabolism-related pathways in MEN and TL tissue cells (**Figure 4D**). To gain further insight, we conducted a more detailed visualization analysis of some metabolic pathways. We used violin plots to examine the expression levels of “oxidative phosphorylation”, “glutathione metabolism”, “glycosaminoglycan degradation”, and “glycolysis/gluconeogenesis” in the four cell subpopulations, two groups, and cell cycle phases (**Figures 4E–G**). We found that the C2 subpopulation exhibited relatively higher expression levels in these pathways, while “glycosaminoglycan degradation” and “glycolysis/gluconeogenesis” pathways were more highly expressed in MEN tissue. This suggested that “glycosaminoglycan degradation” and “glycolysis/gluconeogenesis” might be linked to MEN progression, potentially promoting tumor initiation and development through specific mechanisms. Based on these findings, we inferred that the metabolic pathways in MEN might exert their tumor-promoting effects predominantly during the S phase. Additionally, we analyzed the distribution patterns of these four metabolic pathways using contour plots (**Figure 4H**).

### Pseudotemporal trajectory evolution analysis of EC subpopulations

**Figure 5:**
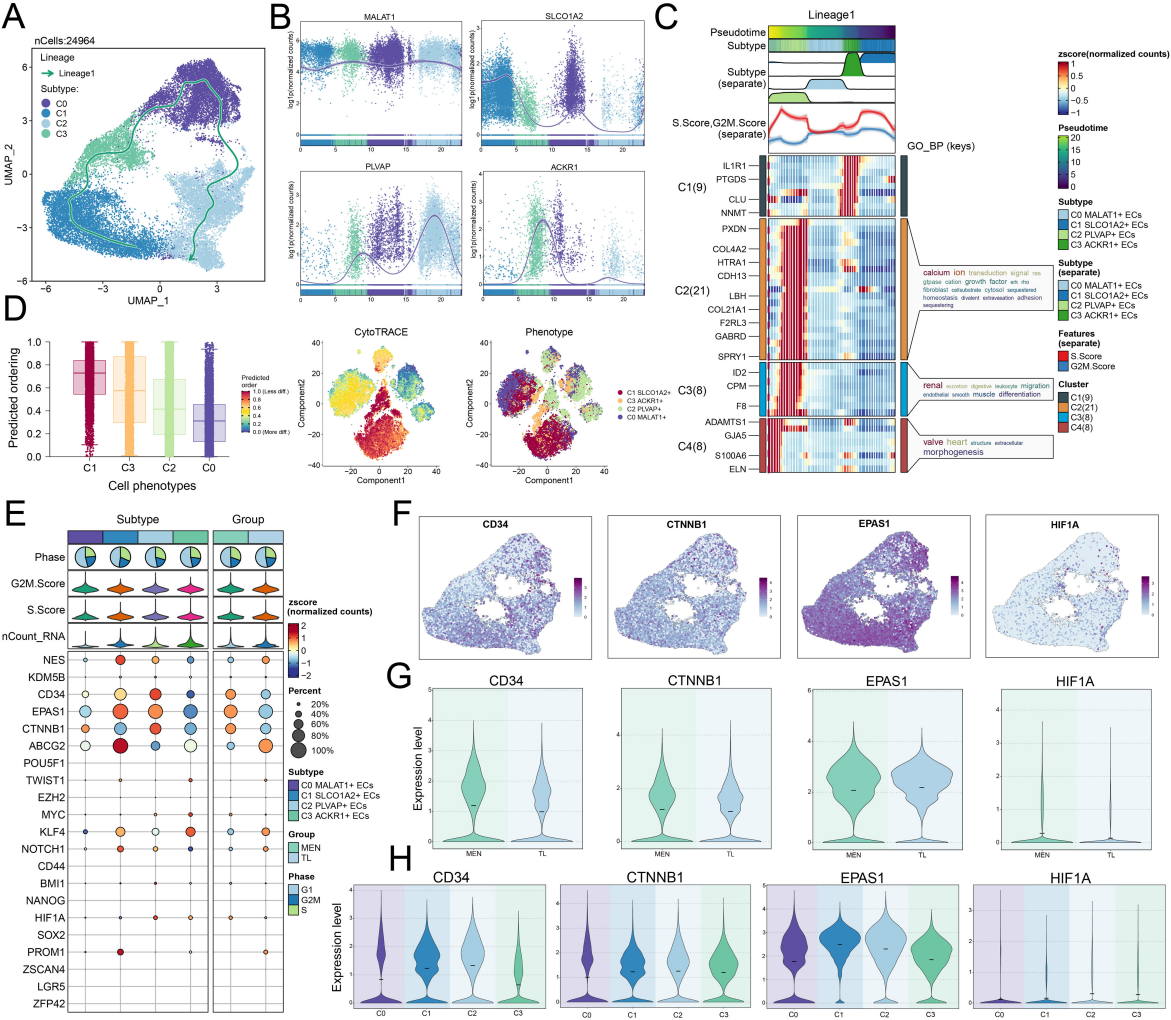
Pseudotime analysis of EC subpopulations. **(A)** UMAP plot showed the pseudotemporal trajectory of lineage 1. **(B)** Dynamic trend plots illustrated the temporal changes of the four EC subpopulations over time. **(C)** Heatmap displayed the DEGs across different cell clusters, along with the enriched GOBP terms. **(D)** Inferred stemness order of EC subpopulations using CytoTRACE (left). The right plot showed the stemness scores of cell subpopulations and their corresponding cell types. **(E)** Bubble plot showed the expression levels of stemness-related genes across different cell subpopulations and tissues. **(F)** UMAP plots visualized the distribution expression of *CD34*, *CTNNB1*, *EPAS1*, and *HIF1A* genes within EC subpopulations. **(G, H)** Violin plots showed the expression levels of *CD34*, *CTNNB1*, *EPAS1*, and *HIF1A* genes across different tissues (MEN and TL) and subpopulations.

After identifying ECs and the C2 subpopulation, we performed a pseudotemporal analysis to validate the screening results. The UMAP plot illustrated the overall evolutionary trajectory of 24,964 cells (**Figure 5A**). The plot showed the lineage trajectory of lineage 1 over time, with differentiation sequence from C1 → C3 → C0 → C2 (“→” represented the differentiation order). As mentioned earlier, the C1 subpopulation mainly originated from TL tissue cells, which were composed mainly of normal tissue cells, while the C2 subpopulation predominantly came from MEN tissue cells. The evolution from normal tissue to pathological tissue adhered to the general disease progression pattern. Next, we performed dynamic trend analysis of the marker genes in each subpopulation. The results showed that the expression level of *MALAT1* remained relatively stable, *SLCO1A2* were highly expressed in the early stages, *PLVAP* were highly expressed in the later stages, and *ACKR1* showed higher expression in the mid-stages. These dynamic trends were consistent with the disease progression lineage in the Slingshot analysis (**Figure 5B**). To gain a clearer understanding of the progression of different subpopulations in disease development, we clustered genes with similar functions into four clusters and visualized their expression levels over time using heatmap (**Figure 5C**). In addition, we used CytoTRACE to predict the ordering of the subpopulations (**Figure 5D**). We found that the C1 subpopulation scored the highest, followed by the C3 subpopulation, and then the C2 subpopulation. The C2 subpopulation might possess enhanced stemness and contribute to tumor progression.

In addition, we analyzed the stemness genes of ECs subpopulations and the TL and MEN tissues (**Figure 5E**). The results showed that genes such as *CTNNB1* were highly expressed in the C0 subpopulation, while *NES, CD34, EPAS1, ABCG2, KLF4*, *NOTCH1*, and *PROM1* were highly expressed in the C1 subpopulation. In the C2 subpopulation, *NES*, *CD34*, *EPAS1*, *CTNNB1*, and *HIF1A* showed high expression, and in the C3 subpopulation, genes like *TWIST1*, *MYC*, *KLF4*, and *HIF1A* were highly expressed. In MEN tissue, genes such as *CD34*, *EPAS1*, *CTNNB1*, and *HIF1A* were highly expressed, while in TL tissue, *NES, ABCG2, KLF4*, and *NOTCH1* were more highly expressed. By observation, we found that the gene expression in the C2 subpopulation closely resembled that of MEN tissue, prompting a deeper analysis of these shared genes. We visualized the distribution of *CD34*, *CTNNB1*, *EPAS1*, and *HIF1A* genes in the UMAP plots (**Figure 5F**). Using violin plots, we visualized the expression levels of these stemness genes across different tissues and subpopulations. The expression levels of these stemness genes were higher in MEN tissue than in TL tissue except *EPAS1*, and these genes showed relatively higher expression in the C2 subpopulation (**Figures 5G, H**).

### Intercellular communication effects

**Figure 6:**
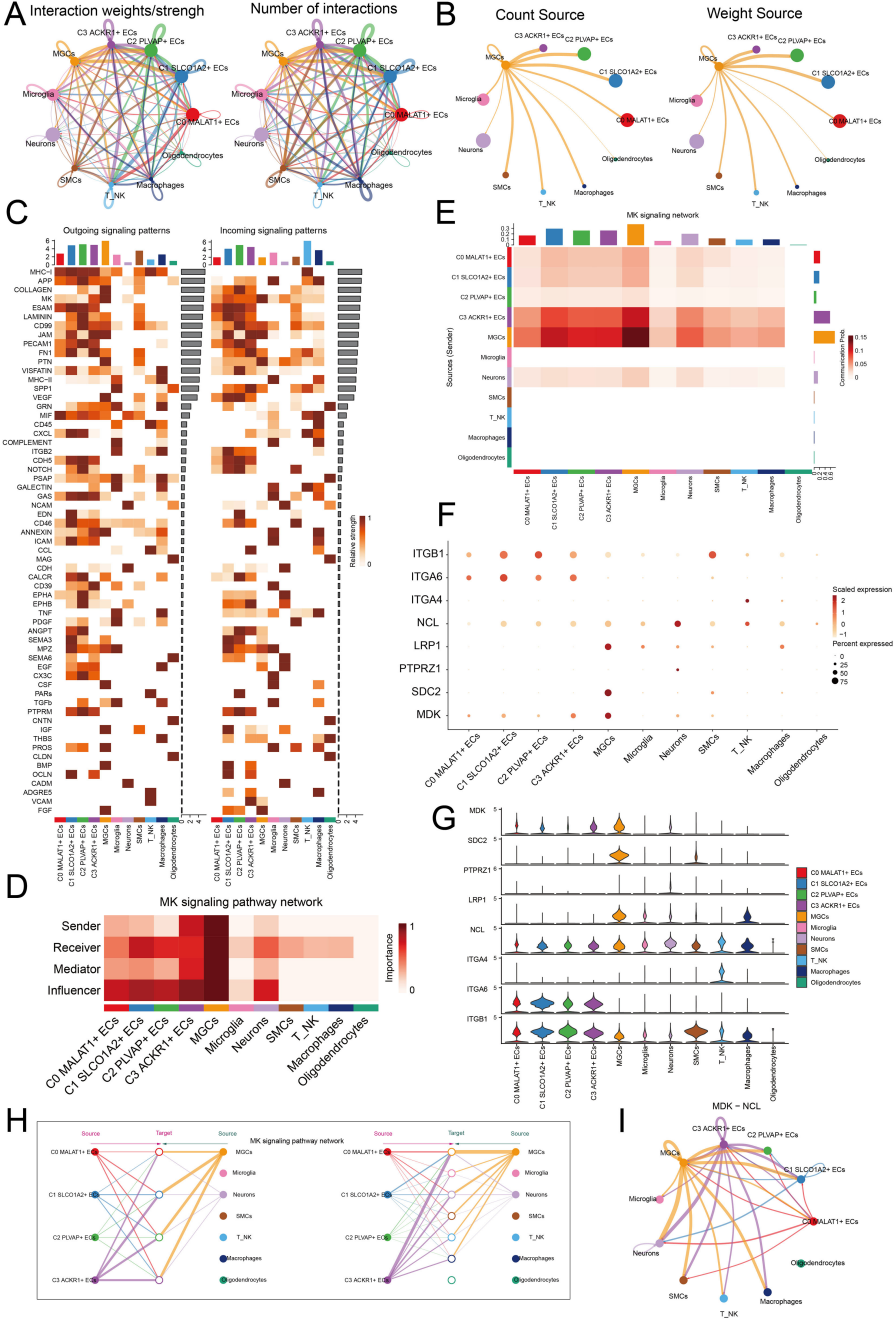
Cellular communication network analysis. **(A)** The interaction intensity (left) and quantity (right) between different cell types were comparatively analyzed. **(B)** MEGs were analyzed as a signal source to observe its communication count (left) and weight (right) with other cells. **(C)** Heatmaps were used to compare the relative strength of outgoing and incoming signaling patterns among different cell types. The bar charts above the heatmap represented the distribution of cell types. **(D)** The roles of different cell types in the MK signaling pathway network were comparatively analyzed. **(E)** The communication probabilities of different cell types within the MK signaling pathway network were analyzed. A heatmap with bar charts above and to the right represented various cell types, where darker colors indicated stronger communication probabilities. **(F)** The expression levels of ligand-receptor pairs in different cell types were comparatively analyzed. **(G)** Violin plots were used to illustrate the expression levels of ligands and receptors in different cell types. **(H)** The signal crosstalk among different cell types within the MK signaling pathway network was comparatively analyzed. **(I)** The role of the MDK-NCL ligand-receptor pair in a circle plot was analyzed.

To further elucidate the crosstalk among TME, we analyzed the intensity and quantity of interactions between EC subpopulations and other cell types using circle diagrams (**Figure 6A**). We found that interactions between MGCs and the C2 *PLVAP*+ ECs subpopulation was particularly significant. When considering MGCs as signal senders, both the interaction counts and weight revealed substantial connections to the C2 *PLVAP*+ ECs subpopulation (**Figure 6B**). This suggested that MGCs could influence the C2 *PLVAP*+ ECs subpopulation via specific signals, thereby stimulating certain biological processes in C2 *PLVAP*+ ECs that promoted the development of MGCs. Next, we used heatmaps to analyze the relative strength of outgoing and incoming signaling patterns across cell types (**Figure 6C**). MGCs and the C2 *PLVAP*+ ECs subpopulation exhibited notable communication in the MK pathway. Moreover, a centrality score heatmap revealed the roles of various subpopulations within the MK signaling pathway (**Figure 6D**). MGCs functioned as senders, receivers, mediators, and influencers, while the C2 *PLVAP*+ ECs subpopulation predominantly acted as receivers and influencers. These findings were corroborated by **Figure 6E**, which demonstrated the communication probability between MGCs and the C2 *PLVAP*+ ECs subpopulation within the MK signaling pathway network.

Subsequently, we examined ligand-receptor pairs involved in this signaling pathway (**Figures 6F, G**). The ligand MDK was highly expressed in MGCs, while the receptor NCL was expressed in the C2 *PLVAP*+ ECs subpopulation. We speculated that the two cell types primarily engaged in crosstalk via the MDK-NCL ligand-receptor pair. EC subpopulations might provide developmental conditions for MGCs through MDK-NCL interactions. MGCs transmitted signals to the C2 *PLVAP*+ ECs subpopulation, enabling ECs to exhibit tumor-promoting effects. A hierarchical graph illustrated the modes of interaction between cell types in the MK signaling pathway (**Figure 6H**). We observed substantial communication between MGCs and ECs, suggesting a complex relationship. This aberrant signaling likely disrupted normal biological processes, potentially contributing to the aberrant development of MGCs. Finally, the circle plot highlighted the relationship between the two cell types, showing that MGCs and the C2 *PLVAP*+ ECs subpopulation interacted via the MDK-NCL ligand-receptor pair (**Figure 6I**). MDK-NCL played a crucial role in EC angiogenesis, and the abundant vasculature it facilitated provided favorable conditions for MEN progression.

### TFs analysis of EC subpopulations

**Figure 7:**
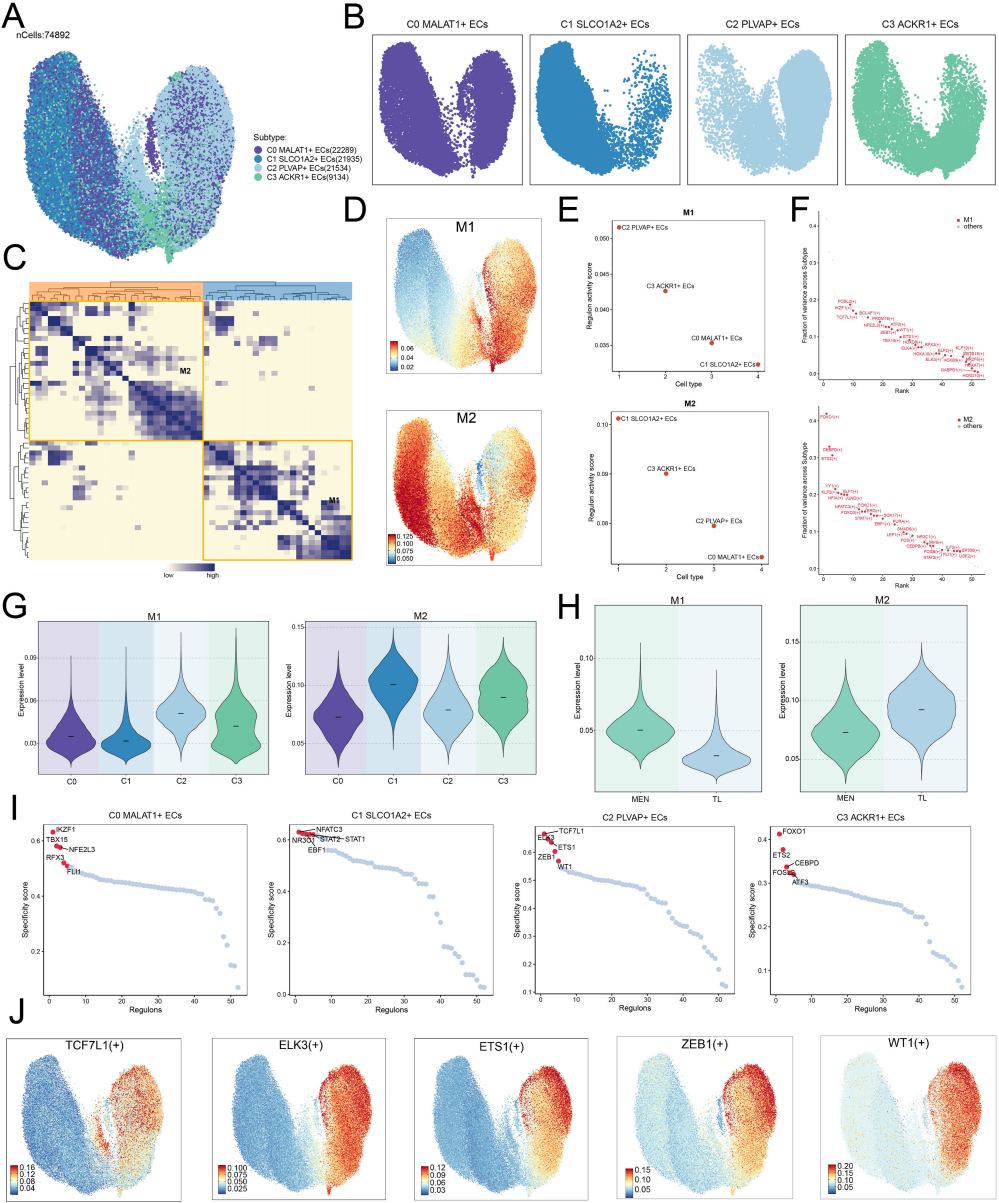
TFs regulatory mechanisms in EC subpopulations. **(A, B)** UMAP plots illustrated the distribution of TFs across EC subpopulations. The UMAP facet plots on the right showed the specific distribution of TFs in each subpopulation. **(C)** Heatmap displayed the clustering analysis of TF correlations, revealing two modules: M1 and M2. **(D)** UMAP plots showed the expression levels of TFs in the M1 and M2 modules. **(E)** Regulon activity scores for different EC subpopulations within the M1 and M2 modules. **(F)** Variance contribution distribution of TFs in different subpopulations, categorized into M1 and M2 modules based on their characteristics. **(G, H)** Violin plots illustrated the expression levels of the M1 and M2 modules across different subpopulations and tissues (MEN and TL). **(I)** Scatter plots showed the specificity scores of the top 5 TFs in each subpopulation. **(J)** The UMAP plots showed the transcriptional activities of the top 5 TFs derived from the C2 subpopulation in the M1 module.

After selecting the cell subpopulations, we further investigated the TFs in ECs. We re-clustered the TFs of each subpopulation and displayed their distributional characteristics using UMAP plot (**Figure 7A**). We then analyzed the distributional characteristics of TFs in each subpopulation (**Figure 7B**). TFs with similar or synergistic functions were grouped into two distinct modules: M1 and M2 (**Figure 7C**), and the distribution patterns of these modules were analyzed using UMAP plots (**Figure 7D**).

We found that in the M1 module, the C2 subpopulation had a higher regulon activity score, while in the M2 module, the C1 subpopulation had a higher regulon activity score (**Figure 7E**). Since the C2 subpopulation predominantly originated from MEN tissue, the TFs associated with the M2 module might facilitate tumor cell proliferation and dissemination through certain mechanisms. Next, we discovered that TFs in the M1 module, such as FOSL2 (+), IKZF1 (+), and TCF7L1 (+), contributed significantly to variance across subpopulations, suggesting that these TFs might play a key role in M1-related biological processes. Similarly, TFs in the M2 module, such as FOXO1 (+), CEBPD (+), and ETS2 (+), also contributed significantly to variance, indicating their importance in M2-specific functions or signaling pathways. These results revealed the modular characteristics of the TF regulatory network (**Figure 7F**).

Additionally, we visualized the expression levels of the M1 and M2 modules in different subpopulations and groups (**Figures 7G, H**). As expected, the expression levels of the M1 module were higher in the C2 subpopulation and MEN tissue, while the M2 module showed higher expression levels in the C1 subpopulation and TL tissue. We also analyzed the specificity scores of the TFs in each subpopulation (**Figure 7I**). Given the C2 subpopulation’s higher potential to drive disease progression, we focused on analyzing the TFs in the C2 subpopulation. The top 5 TFs in the C2 subpopulation were further analyzed using UMAP plots to explore their activity scores (**Figure 7J**).

### *In vitro* experiments related to TF ETS1

**Figure 8:**
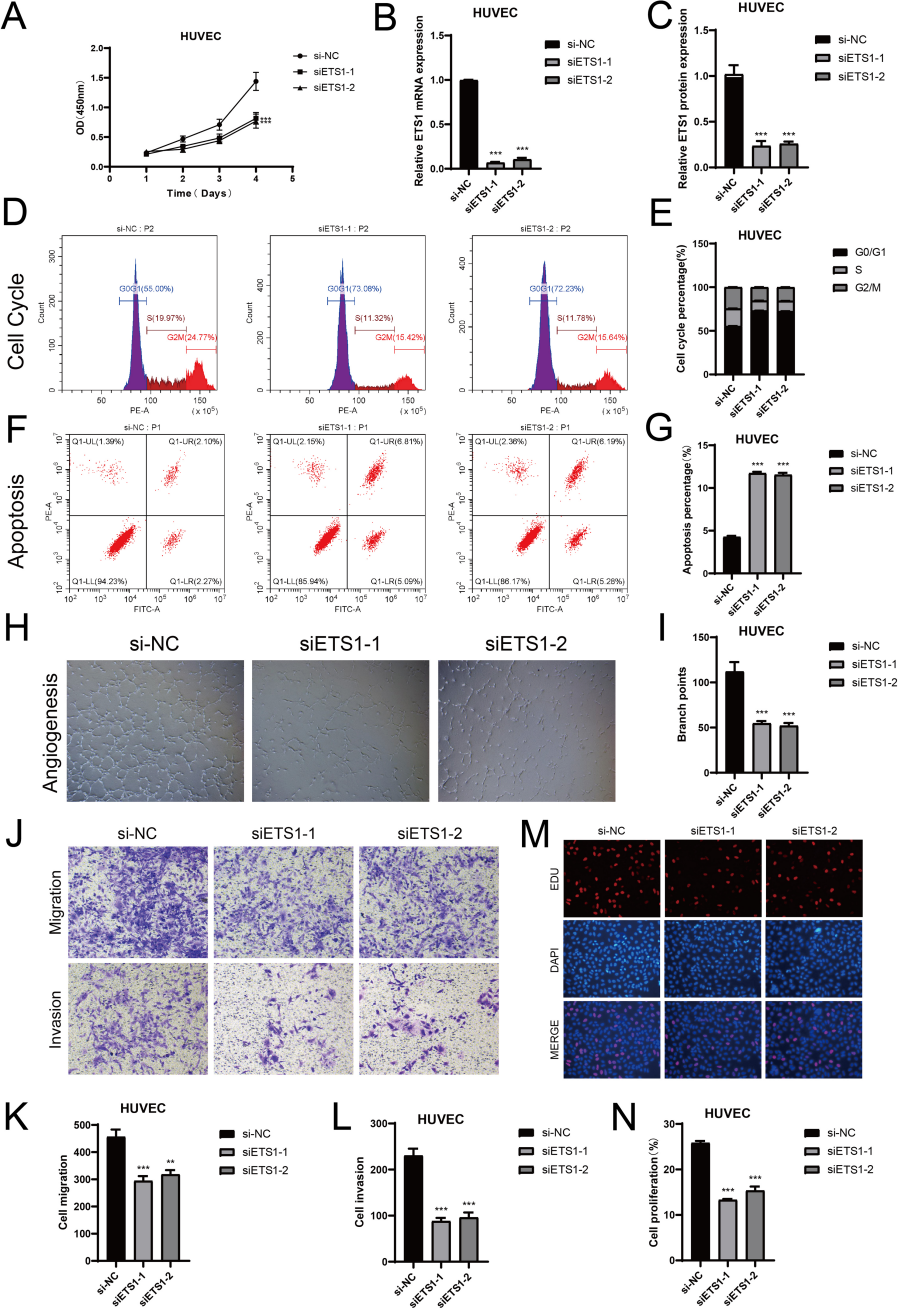
Knockout of ETS1 resulted in changes in ECs *in vitro* experiments. **(A)** The comparison of cell viability before and after ETS1 knockout. **(B, C)** The mRNA and protein levels in ECs were analyzed, both prior to and following ETS1 knockout. **(D, E)** Compare the changes in cell cycle phases (G0/G1, S, G2/M) both prior to and following ETS1 knockout. **(F, G)** Changes in apoptosis rate following ETS1 knockout. **(H, I)** The results of tube formation assays verified the functional changes of ECs before and after ETS1 knockout. **(J-L)** After ETS1 knockout, transwell assays demonstrated a notable reduction in HUVECs’ migration and invasion. **(M, N)** After ETS1 knockout, EdU staining demonstrated that HUVECs’ proliferation was suppressed. (**P < 0.01, ***P < 0.001).

To further clarify the potential relationship between meningiomas and endothelial cell subsets, we conducted *in vitro* experiments. In the C2 subpopulation, ETS1 had a strong activity score. To further validate the role of ETS1 in ECs, we conducted *in vitro* experiments. First, we used the CCK8-assay to observe the changes in the viability of HUVECs before and after ETS1 knockout. The results showed that the cell viability significantly decreased after ETS1 knockout (**Figure 8A**). Then, we carried out a more in - depth study on the mRNA and protein expression levels related to before and after ETS1 knockout (**Figures 8B, C**). Compared with the negative control group, the mRNA and protein expressions in HUVECs after ETS1 knockout were lower. Thus, we concluded that ETS1 could affect the viability of ECs and promote the progression of related biological processes of ECs to some extent. In addition, to further explore the effects of ETS1 knockout on ECs’ proliferation, we investigated the cell cycle phases of HUVECs (**Figures 8D, E**). We found that after ETS1 knockout, the proportion of HUVECs in the G0/G1 phase was higher. In comparison, the ECs in the negative control group had stronger proliferation ability. In the cell apoptosis experiment (**Figures 8F, G**), we obtained similar results. The proportion of apoptosis was higher after ETS1 knockout, which affected EC proliferation to a certain extent. The subsequent tube formation assays further supported this view (**Figures 8H, I**). We could observe significant changes in branch points before and after ETS1 knockout. According to the transwell assays, we learned that ETS1 also played an important role in the migration and invasion processes of ECs (**Figures 8J–L**). After ETS1 knockout, both the migration and invasion of ECs were inhibited. We reached a similar conclusion in the EdU assays (**Figures 8M, N**). There were more cells and more significant fluorescence staining in the negative control group, and the cell proliferation was lower after ETS1 knockout.

In conclusion, ETS1 could promote angiogenesis, and the poor prognosis of meningioma was mainly due to abundant blood vessels. The experiment verified that ETS1 knockout could significantly inhibit angiogenesis. Then we speculated that ETS1 knockout could inhibit the progression of meningioma. Targeting ETS1 became a potential direction for the treatment of meningioma.

## Discussion

Existing studies showed that the incidence of MEN was steadily increasing (86). Although most MEN was benign tumors, they still presented numerous challenges during treatment. According to the available literature, MEN exhibited significant heterogeneity in both onset and treatment (87). To delve deeper into the pathogenesis of MEN, we utilized scRNA-seq technology to study this disease. Initially, we analyzed the obtained single-cell dataset, selected high-quality cells, and performed dimensionality reduction clustering to classify these cells into eight distinct types. Among them, ECs drew our attention. Existing research indicated that ECs played a role in the development of various tumors (88, 89). ECs contributed to tumorigenesis and progression by promoting angiogenesis, shaping the TME, supporting tumor metastasis, and causing abnormal tumor vasculature (90–92). They might provide nutritional and metabolic support through mechanisms such as the VEGF signaling pathway, and assisted tumor metastasis through immune evasion and barrier disruption. Moreover, abnormalities in the structure and function of tumor vasculature not only exacerbated malignancy but also impacted drug delivery and therapeutic efficacy (16). Therefore, we focused on the ECs cluster and conducted further analysis, subdividing it into four subpopulations. These subpopulations’ marker genes were directly or indirectly involved in angiogenesis. We discovered that the C2 *PLAVP*+ ECs subpopulation predominantly originated from MEN tissue. Existing research had shown that *PLVAP* was a membrane protein unique to ECs, primarily distributed in small blood vessels and capillary openings, as well as in fenestrated vesicles (93). It regulated material exchange across the vascular wall, supporting dynamic adjustments of endothelial permeability, thus promoting the maturation and functionalization of newly formed vessels. *PLVAP* played a crucial role in angiogenesis by enhancing EC migration and proliferation (94). A rich vascular network provided essential nutrients and oxygen to tumor cells, promoting their proliferation and growth. Therefore, we may speculate that targeting *PLVAP* therapy is expected to be a potential therapeutic molecule to inhibit the development of meningioma.

In our metabolic analysis, we found that biological processes such as oxidative phosphorylation, glutathione metabolism, and glycolysis/gluconeogenesis exhibited higher AUCell scores in the C2 subpopulation. This suggested that these biological processes were more active or strongly expressed in the C2 subpopulation, playing a critical role in angiogenesis and tumor progression (95–97). These processes supported tumor rapid growth, invasion, and metastasis by regulating energy metabolism, redox states, and changes in the microenvironment. Particularly in hypoxic microenvironments, enhanced glycolysis promoted lactic acid accumulation, which could activate angiogenesis signaling pathways (98). Furthermore, the regulation of redox balance through glutathione metabolism helped stabilize and form vasculature within the TME (99).

Additionally, we utilized Slingshot and CytoTRACE to assess the differentiation process of each subpopulation in the disease. The C2 subpopulation was found to be at the terminal differentiation stage, which could play a key role in disease progression. To further clarify the underlying mechanisms, we analyzed stemness-associated genes across various cell types. We observed that the gene expression profiles of the C2 subpopulation closely resembled those of MEN tissues. *CD34*, a cell surface marker primarily expressed in endothelial progenitor cells, hematopoietic stem cells, and fibroblasts, played an important role in EC proliferation, migration, and lumen formation during angiogenesis (100). *CTNNB1* encoded β-catenin, which regulated the Wnt signaling pathway, an important factor in angiogenesis (101). β-catenin promoted the formation of new blood vessels by regulating EC proliferation, differentiation, and migration (102, 103). Under hypoxic conditions, both *EPAS1* (HIF-2α) and *HIF1A* (HIF-1α) were upregulated, leading to the activation of genes associated with angiogenesis (104). Specifically, *HIF-1α* activated the expression of angiogenic factors such as VEGF and angiopoietin, promoting the formation of new blood vessels (105, 106). *EPAS1* (HIF-2α) also played a substantial impact on regulating angiogenesis under hypoxia, particularly in chronic hypoxic environments, by activating similar angiogenic factors (107). In conclusion, *CD34*, *CTNNB1*, *EPAS1*, and *HIF1A* could promote MEN progression by regulating angiogenesis. In other words, these stemness genes were expected to become potential therapeutic targets.

During disease progression, cellular signaling crosstalk often occurred. We identified that the C2 subpopulation likely interacted with MGCs through the MK signaling pathway. The MK pathway is a critical factor in cell proliferation, differentiation, migration, and apoptosis, especially in the development of tumors, inflammation, and cellular senescence (108, 109). In this study, we explored the major ligands and receptors of the MK signaling pathway, such as MDK-NCL, which were involved in the progression of various cancers, including lung cancer, breast cancer, liver cancer, gastric cancer, and colorectal cancer (110). They promoted tumor progression by regulating cell proliferation, migration, angiogenesis, and anti-apoptotic processes (111). Therefore, we hypothesized that the MDK-NCL ligand-receptor pair might have played a crucial role in MEN development, contributing to the dysregulation of signaling pathways.

Furthermore, we conducted a transcription factor regulatory analysis to explore how TFs influenced gene expression, thereby affecting cellular biological processes. Among the many TFs, ETS1 attracted our attention. ETS1, a member of the ETS family of TFs, had been shown to play a key role in angiogenesis (112). ETS1 regulated the expression of various angiogenesis-related genes and participated in EC proliferation, migration, and vessel lumen formation (113, 114). ETS1 played a proactive role in angiogenesis, particularly in tumor-associated angiogenesis (115). In the TME of MEN, ETS1 might have further promoted angiogenesis by regulating local hypoxia and inflammation (116). Hypoxia was a potent inducer of angiogenesis, and ETS1 likely enhanced the expression of VEGF and other angiogenic factors by regulating hypoxia-responsive signaling pathways such as HIF-1α (117, 118). *In vitro* experiments also validated the role of ETS1, showing that it promoted EC proliferation, differentiation, and angiogenesis. Therefore, ETS1 could have been a potential therapeutic target in the treatment of MEN, providing valuable guidance for clinical interventions in MEN patients. Although this study conducted in-depth research on the development of meningiomas, there were still some insufficiencies. Firstly, this study mainly relied on public datasets from the GEO database, which, although providing rich information, inevitably suffered from batch effects and data heterogeneity. Therefore, future research should combine multi-center data to further validate the conclusions. Additionally, the study lacked *in vivo* experimental support. Although scRNA-seq revealed the characteristics of the meningioma microenvironment, it was still necessary to conduct experiments through animal models or clinical samples. In the future, animal experiments will be conducted based on these findings. Moreover, although this study identified some potential treatment-related biomarkers, it failed to propose clear drug targets or verifiable therapeutic hypotheses, which somewhat affected its clinical translational value. Therefore, future research should combine drug screening experiments or gene editing techniques to further explore intervention strategies for meningiomas. Recent studies have shown that immune therapy targeting T cell exhaustion showed promising prospects in non-small cell lung cancer (119), while immune therapy for meningiomas was still in the exploratory stage. Combining scRNA-seq to analyze the immune characteristics of the meningioma microenvironment provided new directions for the development of future immune therapy strategies. At the same time, the combination of scRNA-seq technology and personalized immune therapy strategies was expected to provide more precise treatment options for meningioma patients.

## Conclusion

Based on the single-cell characteristics of MEN, we studied the heterogeneity of the TME in MEN. Through further analysis of the EC subpopulations, we found that the C2 subpopulation was significantly presented in MEN and played an important role in angiogenesis and cell signaling crosstalk. We discovered that the MDK-NCL ligand-receptor pair might play a critical role in intercellular communication. Additionally, we found that ETS1 could promote angiogenesis, thereby providing favorable conditions for MEN progression, a finding that was confirmed *in vitro*. These findings are expected to provide potential therapeutic targets for MEN treatment. Although our study contributes to advancing the treatment of MEN patients, there are still limitations. We plan to collect more comprehensive and reliable data and conduct a more in-depth and precise analysis of MEN from the perspective of prognostic models. In addition, we will introduce a larger clinical cohort to validate the findings and enhance the robustness of the prognostic model. At the same time, we plan to conduct *in vivo* experiments to further validate the function of key genes in meningioma angiogenesis through animal models and evaluate their feasibility as potential therapeutic targets. By optimizing predictive models, conducting *in vivo* functional studies, and exploring targeted intervention strategies, we hope to provide stronger support for accurate diagnosis and personalized treatment of meningiomas.

## Data Availability

The original contributions presented in the study are included in the article/supplementary material. Further inquiries can be directed to the corresponding author.
